# Evaluation of osseous integration of titanium orthopedic screws with novel SLA treatment in porcine model

**DOI:** 10.1371/journal.pone.0188364

**Published:** 2017-11-17

**Authors:** Tzu-Hsiang Lin, Hsin-Tai Hu, Hsueh-Chun Wang, Meng-Chian Wu, Shu-Wei Wu, Ming-Long Yeh

**Affiliations:** 1 Department of Biomedical Engineering, National Cheng Kung University, Tainan, Taiwan; 2 Department of Technology Development, Hung Chun Bio-Science Company, Kaohsiung Science Park, Luzhu, Kaohsiung, Taiwan; 3 Medical Device Innovation Center, National Cheng Kung University, Tainan, Taiwan; Universidad de Zaragoza, SPAIN

## Abstract

The success of many endosseous implants in orthopaedic and dental applications depends on the surface characteristics, as they affect osseous integration. Previous investigations indicated that a novel large-grit sand-blasted and acid-etched (SLA) titanium (denoted as SLAffinity-Ti) implant had better bone integration than that of a comparably shaped implant with a plasma-sprayed titanium surface. The purpose of the present investigation was to create a SLAffinity surface on pedicle screws and trauma screws and to compare it with the surfaces of a sand-blasted-only implant and commercial implants in terms of bone integration. The cortical bone and spine of twelve minipigs were implanted with 3 and 4 implants, respectively, and the bone integration was evaluated using micro-computed tomography (micro-CT), mechanical tests (pull-out strength and stripping torque), and histological analysis (toluidine blue and hematoxylin and eosin staining) one and three months after implantation. The micro-CT images showed that the gap between the bone and implant was consistently higher in the sand-blasted-only and commercial groups compared to that in the SLAffinity group 1 and 3 months after implantation. Moreover, the bone volume of implant inserted into bone and the percentage of implant inside bone tissue were greater in the SLAffinity screws 1 and 3 months after implantation, as compared to the sand-blasted and commercial screws. In the mechanical tests, the removal torque and pull-out strength (*p* < 0.05) were higher in the SLAffinity group at 1 and 3 months. The histological results were consistent with mechanical testing, showing that the SLAffinity group had the most mineralized matrix, the most bone formation around the screws, and the most bone cells in bone tissue. These findings indicate that a SLAffinity surface can effectively enhance the holding strength and integration of pedicle screws and cortical screws, promoting early healing and improving outcomes, compared to sand-blasted-only and commercial implants.

## Introduction

Osteoporosis is a common disease in the elderly. With an aging global population, the proportion of patients undergoing spinal surgery with osteoporotic bone will increase [1]. The prevalence of osteoporosis in US adults has been reported to be in the range of 5% to10% in women and 2% to 4% in men for those 50 years old or over [2, 3]. This presents a serious challenge for spinal surgeons clinically.

Pedicle screw systems have become increasingly common for reconstruction, fixation, and stabilization in spinal disorder surgeries. However, complications, including screw loosening, occur in some cases, especially in patients with osteoporosis. With poor bone mineral density (BMD), inferior bone rigidity decreases the pull-out strength of pedicle screws in osteoporotic bone, eventually leading to screw loosening. In a cortical screw system, screw loosening and subsequent implant failure are the most frequently observed surgical complications [4, 5]. The stability of a fixation mainly depends on the anchorage of the screw in the bone. Several approaches have been proposed, such as locking plates [6] and the cortical bone trajectory [7] technique, to provide considerable fixation strength via fixed-angle stabilization or achieve stronger fixation by increasing the cortical bone purchase within the vertebra, respectively.

An approach to achieve faster and more stable bone integration is implant surface treatment. Common surface modifications include machining, grit blasting, acid etching, alkaline attack, electrochemical methods, and deposition of ions [8]. Previous studies have demonstrated that surfaces with micrometer-scale roughness enhance bone integration compared to that obtained with smooth surfaces. An in vitro study found that an increase in micrometer-scale surface roughness increased osteoblast differentiation and extracellular matrix production, which may give rise to better bone formation and osteointegration in vivo [6, 9]. Large-grit sand blasting and acid etching (SLA) surface treatment has been applied to dental implants for several years and was found to have higher removal torque value (RTV) [10, 11] and bone-to-implant contact [11, 12] compared to no surface treatment group in an animal study and a clinical trial [13]. Pedicle screw systems in clinical application have not yet been treated with SLA.

We previously applied a novel surface modification called SLAffinity to dental implants. With grit-blasted and acid-etched treated by electrochemical functionalization, a SLAffinity-Ti surface had a 500-nm oxide layer deep inside the Ti structure [14]. This structure showed high biocompatibility and wettability, allowing ready adhesion to blood. Growth factors such as PRP or its recombinant growth factor increase the bone maturation rate and improve bone density [15]. Microscale roughness in combination with nanoscale structures and bioactive properties improves osteoblast viability and differentiation [16]. Osseointegration was evident at early stages of bone healing in a pig [17]. SLAffinity-treated dental implants have been shown to be clinically successful over a 4-month period [18].

In this study, we test the hypothesis that SLAffinity treatment on Ti_6_Al_4_V alloy pedicle screws and cortical screws enhances osteointegration and fixation strength at the bone-implant interface compared with those obtained for sand-blasted-only Ti_6_Al_4_V surfaces and commercial smooth screws in a porcine model. The pull-out strength of pedicle screws in a porcine spine and removal torque were measured to evaluate the mechanical integration of the three types of screw surface. Micro-computed tomography (micro-CT) and histological methods were used to observe the integration between bone and screws with different surface treatments.

## Materials and methods

### Cortex and pedicle screws

Commercially available screw-type implants, manufactured from a Ti_6_Al_4_V specimen, were used in this study. The chemical composition of Ti_6_Al_4_V was C (0.08 wt.%), Fe (0.25 wt.%), O (0.13 wt.%), Al (6.75 wt.%), V (4.5 wt.%), and Ti (trace), as described previously [19]. The cortex screws had an external diameter of 3.5 mm and a length of 16.0 mm. The pedicle screws had an external diameter of 4.5 mm and a length of 30.0 mm. Briefly, to prepare SLAffinity Ti_6_Al_4_V specimens, a Ti_6_Al_4_V substrate was used and all specimens were mechanically polished using 1500 grit paper and then further polished using diamond abrasives (1 μm). The specimens were finished with colloidal silica abrasives (0.04 μm). All specimens were degreased by washing in acetone and prepickled in acid, and then processed using 2% ammonium fluoride, a solution of 2% HF, and 10% nitric acid at room temperature for 60 s. Then, the specimens were grit-blasted with Al_2_O_3_ particles. Finally, the specimens were etched in an aqueous mixture of HF and HNO_3_ at room temperature for many seconds and then washed using distilled water in an ultrasonic cleaner. Then, the titanium cortex and pedicle screws were subjected to cathodic polarization at a constant current in 10 M H_2_SO_4_ solution at room temperature. To form the TiO_2_ layer on the Ti_6_Al_4_V surface, titanium was treated using anodization at a constant current of 15 ASD for 10 min in 5 M NaOH solution. For easy identification, Ti_6_Al_4_V screws with sand blasting as pretreatment and SLAffinity treament are denoted as sand-blasted screws and SLAffinity screws, respectively.

### Porcine cortical bone and spine model

All experimental animal procedures were approved by the Animal Care and Use Committee of Chi Mei Medical Center and performed in accordance with strictly aseptic techniques. Twelve healthy male adult Lanyu minipigs (Taitung, Taiwan) weighing between 20 and 30 kg were used in this study. The animals were premedicated with an intramuscular injection of Zoletil and Atropine (2.5 and 0.03 mg/kg, respectively), and a subcutaneous injection of ketorolac (1 mg/kg). Anesthesia was induced with an intramuscular injection of ketamine and diazepam (10 and 0.2 to 0.5 mg/kg, respectively). The animals were connected to a general anesthesia machine that provided isoflurane (2% to 5%) and oxygen (up to 100%). The operations were done with the pigs under general anesthesia and positioned on their abdomen. After the surgical procedure, the animals were monitored daily for signs of inflammation, lameness, and general well-being by an experienced animal care technician and a veterinarian. Post-operative analgesia was achieved with a 75-mg fentanyl dermal patch applied to the back and legs, respectively, and subcutaneous administration of 1 mg/kg ketorolac every 8–12 h for three days. An amount of 10 mg/kg/day antibiotic was administered with food for 10 days post-operation. Animals were allowed free movement with unrestricted weight-bearing activity and housed singly under standard laboratory conditions (temperature of 24°C, 12-hour light-dark cycle) with food and water ad libitum. During the period of the experiment, if the animals exhibited symptoms such as hind limb lameness, generalized muscular tremor, ataxia, or incoordination, they were euthanized humanely. The remaining animals were euthanaized via intravenous injection of sodium pentobarbital overdose after one or three months.

### Implantation of cortex and pedicle screws

Two types of screw, pedicle and cortical screws, were applied in this study. Each type of screw had 3 groups according to the applied surface treatment method. Group A included cortex and pedicle screws with SLA modification (SLAffinity); group B included commercial smooth surface cortex and pedicle screws (Stryker, Taiwan); and group C included smooth cortex and pedicle screws with sand blasting treatment (S1 Fig). The cortex screws (n = 6) were inserted into the cortical bone within the tibia from the hind legs by an orthopedic surgeon using the prescribed instruments to pilot drill, tap, and insert the screws. Pedicle screws (n = 4) were inserted unilaterally and asymmetrically into the pedicular notch of L2 to L5 within the vertebral body. Thus, each pig received three cortex screws per tibia and one pedicle screw per vertebra. The type of screw in each pig was assigned randomly. The implant location of the screws in the hind tibia or the left or right leg and the left or right side of the spinal vertebrae was also randomly assigned (Table 1). Radiographs were made with the animals under general anesthesia to evaluate the placement of the cortex and pedicle screws. Post-operatively, the animals were treated with the nonsteroidal anti-inflammatory drug ketorolac (1 mg/kg) once a day for three days for post-operative pain relief. The animals were able to walk undisturbed after recovery from anesthesia.

**Table 1:** Experimental and control groups.

	Leg		Spinal vertebrae			
Pig No.	Hind		L2	L3	L4	L5
	R	L				
1^&^	A	C	B	A	B	A
2^★^^#^	A	C	C	A	C	A
3^#^	A	C	B	A	B	A
4^&^	A	B	B	A	B	A
5^★^^#^	C	B	C	B	B	C
6^&^	C	B	C	B	B	C
7^&^	C	B	B	B	B	C
8^#^	C	B	B	C	C	B
9^#^	A	B	B	A	A	B
10^&^	A	B	A	B	B	A
11^#^	A	C	A	C	C	A
12^&^	A	C	A	C	C	A

R = right, L = left, A = group A, SLAffinity implant; B = group B, commercial implant; C = group C, sand-blasted implant.

^★^: The pig died 24 hours after surgery.

^#^: The pigs (nos. 2, 3, 5, 8, 9, and 11) were sacrificed at 1 month after surgery.

^&^: The pigs (nos. 1, 4, 6, 7, 10, and 12) were sacrificed at 3 months after surgery.

72 cortex screws (3 implants in each tibia from leg, group A: SLAffinity (n = 24), group B: commercial (n = 21), group C: sand blasting treatment (n = 27)) and 48 pedicle screws (1 implant in each vertebral body, group A: SLAffinity (n = 16), group B: commercial (n = 19), group C: sand blasting treatment (n = 13)) were allocated to each test.

### Assessment of cortex and pedicle screw fixation

The animals were sacrificed 1 and 3 months after screw implantation. The tibia and L2 to L5 vertebrae were removed with the screws en bloc. An individual tibia and vertebra from each leg and spinal section (L2-L5) had 1 tibia and a minimum of 1 vertebral body (S2 Fig) allocated to each test, respectively. The tibia and left and right vertebrae were imaged using micro-CT, and then 48 vertebral bodies (group A: SLAffinity (n = 16), group B: commercial (n = 19), group C: sand blasting treatment (n = 13)) were allocated for pull-out testing and 24 tibias (1 leg containing 3 implants, group A: SLAffinity (tibia, n = 8; implant, n = 24), group B: commercial (tibia, n = 7; implant, n = 21), group C: sand blasting treatment (tibia, n = 9; implant, n = 27)) were allocated for the torsion test and histological analysis, respectively. For histological analysis, one tibia from each animal was fixed in 4% buffered formalin and processed for undecalcified bone histomorphometry, as described below. The other tibia and L2 to L5 vertebrae were frozen in saline solution and stored at -80°C for later micro-CT imaging and mechanical testing. After thawing, vertebrae were examined with micro-CT and then used for the pull-out test. Tibia were used for the analysis of removal torque. Mechanical testing and histomorphometry were performed only on the osteointegrated screws, including malpositioned screws that had contact with bone, as determined from radiographic analysis. Malpositioned screws that failed to become at least partially integrated into the host bone were not included in the statistical analysis, based on the assumption that the failure to support bone formation was a surgical failure or was specific to a particular animal and not due to a surface property.

### Radiographic and micro-CT analysis

After thawing, each tissue block from L2 to L5 vertebrae was scanned with a voxel spacing of 35 μm (Skyscan 1076; Bruker Micro CT, Belgium). Although computer software is available for subtracting the metallic implant from bone, the scatter caused by the metal screws was too strong to permit the contrast needed to determine the bone-implant contact at the interface with the use of micro-CT. However, the images were useful for a general assessment of osteointegration. Therefore, after scanning, the area of the bone-screw interface of the specimen was quantified by densitometric analysis using videomicroscopy and ImageJ software (National Institutes of Health); the signals were normalized relative to the control group. On the other hand, the CT images were used to measure the bone-volume percentage, which is the percentage of mineralized bone in the total tissue volume [20]. In addition, the percentage of pedicle screws inside bone tissue was also evaluated by densitometric analysis using CT images.

### Mechanical testing

**Fig 1:**
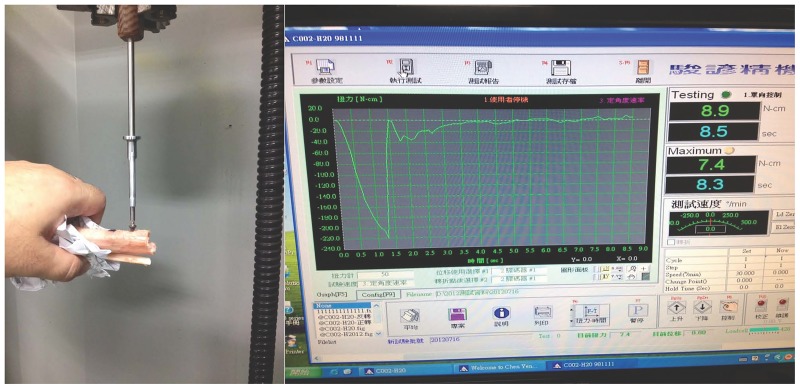
Digital torque gauge used to determine RTV of cortical screws.

The removal torque of cortex screws was measured using a calibrated MTS 858 Mini Bionix II testing system (MTS, Eden Prairie, Minnesota) (Fig 1). Tissue blocks from the tibia for 28 and 21 cortex screws, obtained one and three months after implantation, respectively, were tested. The exposed end of the screw was attached to an adapter fixed to the axial-torsional load transducer. The screws were rotated 60° counterclockwise at a rate of 0.5°/s. The torque angle and moments were recorded automatically with a TestStar data acquisition system (MTS). From the torsion tests, the maximum torque and angle stiffness were calculated. The maximum torque was defined as the removal torque.

**Fig 2:**
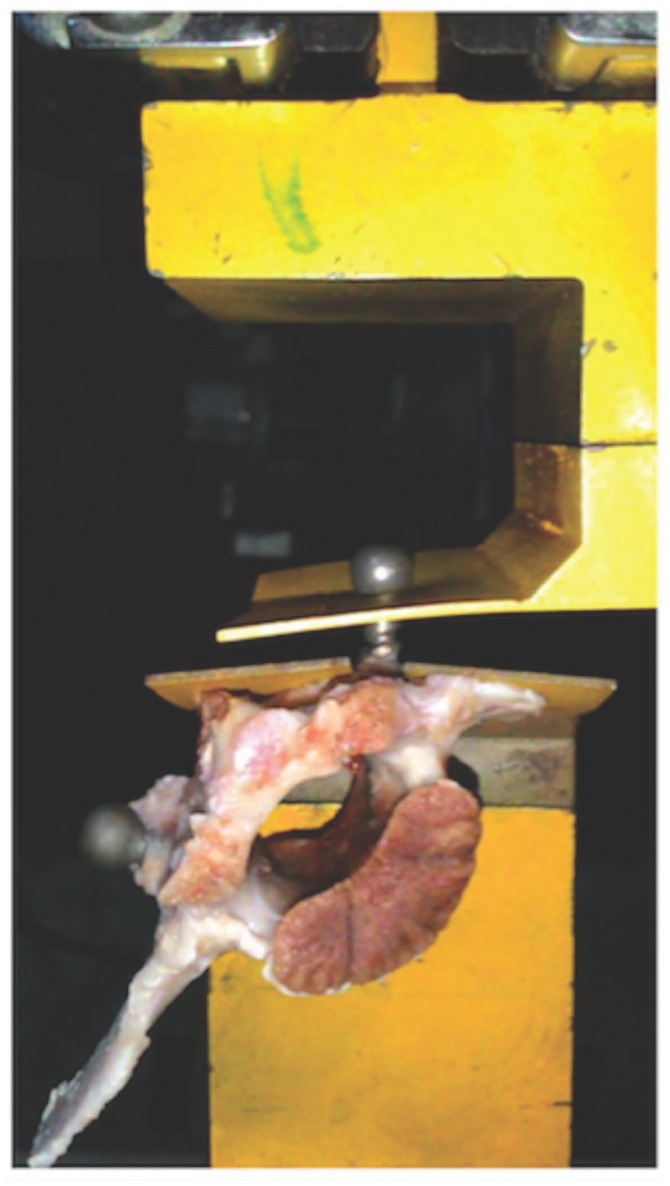
Setup of material testing system for pull-out test of pedicle screws.

The goal of the pull-out test of pedicle screws was to measure the axial pull-out strength for the failure of pedicle screw fixation. Each specimen was mounted onto a material testing system (Hung Ta HT-2402BP, Taichung, Taiwan) through a custom-made clamping device (Fig 2). The pedicle screw orientation among specimens was adequately adjusted to keep the alignment of the applied pulling force. The pedicle screw was attached to the apparatus by a thick sheet with a round hole to secure the screw head. A pre-load of 10 N for 30 s was applied first for ensuring that the structure was stabilized to prevent a sudden impact may occur at the initiation of the pull-out test. Then, a continuous and progressive displacement at a rate of 5 mm/min was exerted for pulling out the pedicle screw following the guidelines of ASTM F543-13. The peak force was recorded as the pull-out strength (maximal pull-out force).

### Histological analyses

Tissue sections were prepared from plastic-embedded formalin fixed tissue blocks with the implant intact from the tibia 3 months after implantation. These sections were ground to a final thickness of 10 to 20 μm and stained with hematoxylin and eosin (H&E) for qualitative histological analysis. Non-osteointegrated screws were not included in the histological assessment described below because bone-implant contact was not present and therefore could not be evaluated. Moreover, the tissue sections were also stained with toluidine blue to detect the bone characteristics from the bone formation between bone and implant. Examinations were performed with a light microscope (Axiophot-2, Zeiss, Jena, Germany) equipped with a digital camera (DC 500, Leica, Bensheim, Germany) for descriptive evaluation.

### Statistical analyses

Statistical analyses were performed using the SPSS v17.0 software package. All of the data are shown as the mean ± standard error of mean (SEM). Because the data were not normally distributed, the homogeneity of variance was corroborated using Levene’s test. The nonparametric Kruskal-Wallis test followed by the Mann-Whitney U-test were used to analyze the data among the three groups at each time point for torque force and pull-out strength, respectively. A *p*-value of < 0.05 with a 95% confidence interval was defined as a significant difference.

## Results

The average weights of the pigs in all three groups were at 21.98 ± 2.88 kg. There were no apparent differences between the three groups in terms of weight. The two animals that died during surgery or the next day seemed to be unable to withstand anesthesia. The remaining animals recovered from anesthesia without complications and had good wound healing. The screws were well positioned in the tibia and L2 to L5 vertebral segments. At every indicated time interval, tissue blocks with the screws were collected. None of the pigs showed any signs of substantial weight loss or gain.

### Radiologic evaluation

**Fig 3:**
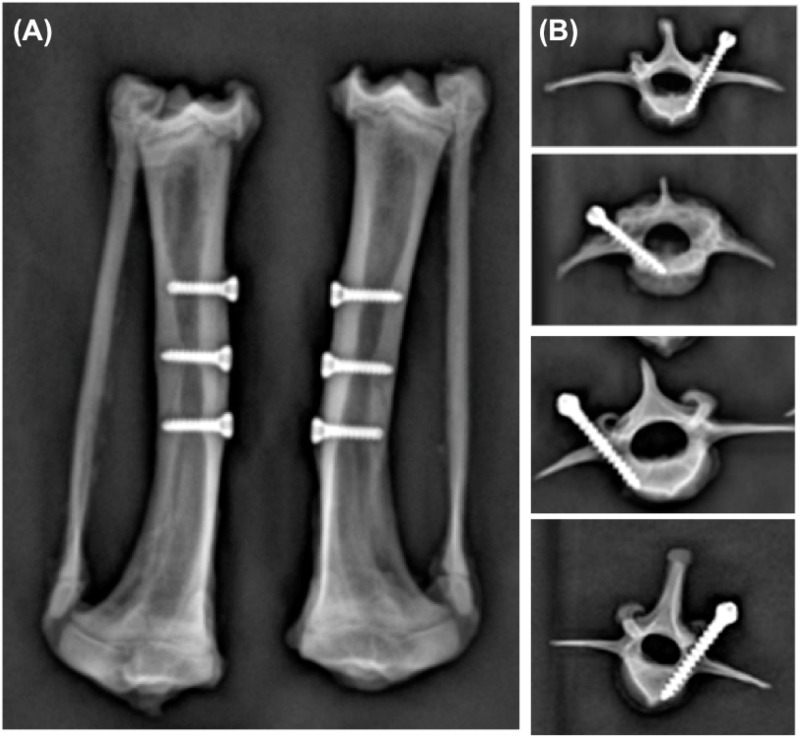
Representative radiographs showing cortex and pedicle screws after implantation. (A) Cortex screw with cortical bone placement and (B) Pedicle screw with traditional placement on L2-L5 vertebrae.

**Fig 4:**
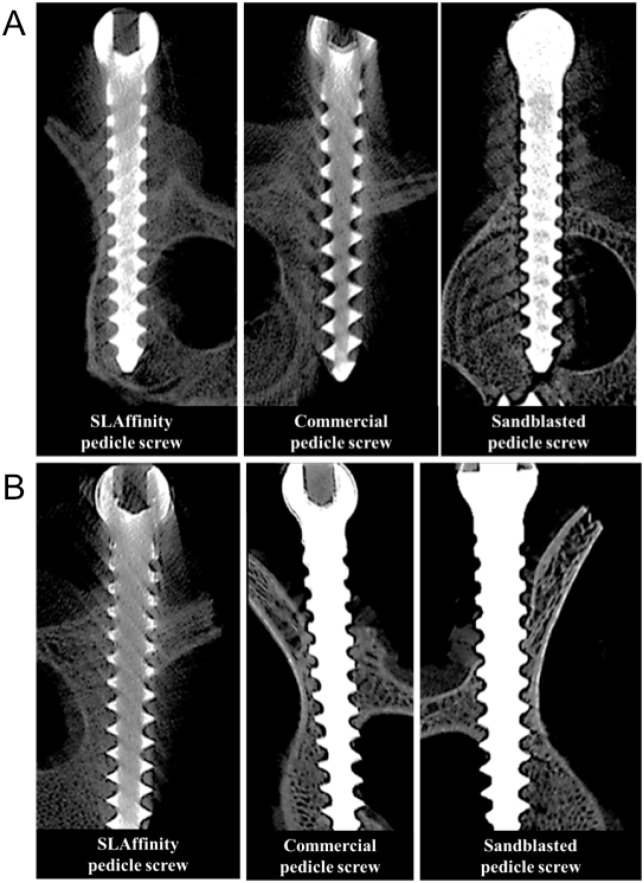
Micro-CT images of different pedicle screws inserted into vertebral column after implantation. (A) 1 month and (B) 3 months after implantation.

**Fig 5:**
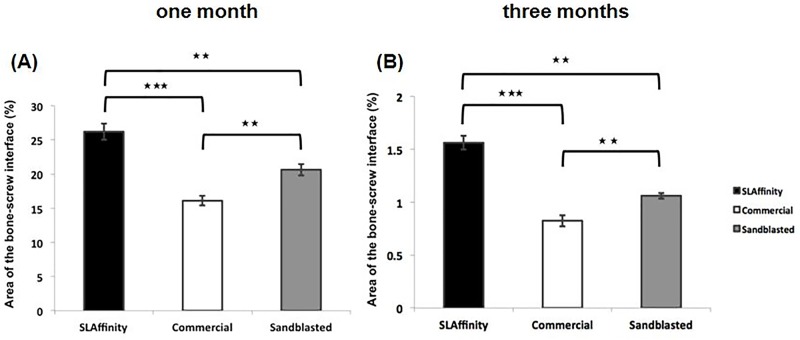
Quantitation of micro-CT images of different pedicle screws inserted into vertebral column after implantation. (A) 1 month and (B) 3 months after implantation. *: *p* < 0.05,**: *p* < 0.01, and ***: *p* < 0.001.

**Fig 6:**
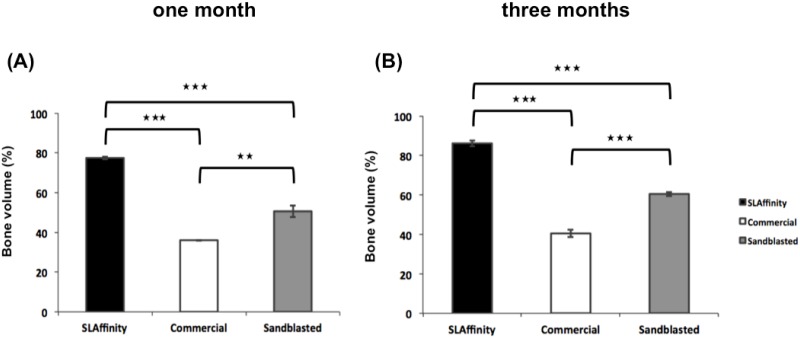
The bone volume of different pedicle screws inserted into vertebral column after implantation. (A) 1 month and (B) 3 months after implantation. **: *p* < 0.01 and ***: *p* < 0.001.

**Fig 7:**
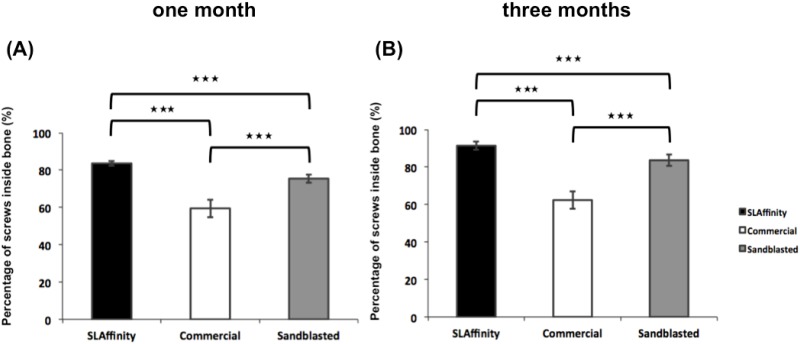
The percentage of different pedicle screws inside vertebral column after implantation. (A) 1 month and (B) 3 months after implantation. ***: *p* < 0.001.

Radiographs showed that the screws remained firmly within the bone of the tibia (Fig 3A) and L2 to L5 vertebrae (Fig 3B) after implantation. These results of pedicle screws were confirmed using micro-CT. Major bone formation and a minor gap of the peri-implant was found in the SLAffinity group compared to those of the other groups 1 and 3 months after implantation, respectively. Moreover, the sand-blasted group presented more osseous production and a smaller gap at the bone-screw interface compared to those of the commercial implants (Fig 4A and 4B). However, the images of the implant-bone interface were distorted due to scattering by the metal implant, preventing a quantity assessment of the bone tissue at the interface. Thus, the quantitation of radiographic images was performed using densitometric analysis. The area of the bone-screw interface of the specimens obtained 1 and 3 months after implantation were evaluated, respectively. The results indicate that the SLAffinity group had a larger interface between screws and bone tissues compared to those of the sand-blasted (1 month, *p* = 0.002; 3 months, *p* = 0.002) and commercial groups (1 month, *p* < 0.001; 3 months, *p* < 0.001) 1 and 3 months after implantation, respectively. Furthermore, the sand-blasted group presented a larger area of the bone-screw interface compared to that of the commercial group (1 month, *p* = 0.001; 3 months, *p* = 0.002) 1 and 3 months after implantation, respectively (Fig 5A and 5B). Results from bone volume analysis showed more mineralized tissue formation in the area of peri-implant bone in SLAffinity group compared to those of the sand-blasted (1 month, p < 0.001; 3 months, p < 0.001) and commercial groups (1 month, p < 0.001; 3 months, p < 0.001) 1 and 3 months after implantation, respectively (Fig 6A and 6B). In addition, the percentage of screw inside bone tissue was greater for the SLAffinity screws: 83.6% compared with 75.4% and 59.4% for the sand-blasted (p < 0.001) and commercial (p < 0.001) screws 1 month after implantation, respectively and 91.4% compared with 83.6% and 62.3% for the sand-blasted (p < 0.001) and commercial (p < 0.001) screws 3 months after implantation, respectively (Fig 7A and 7B).

### Mechanical tests

**Fig 8:**
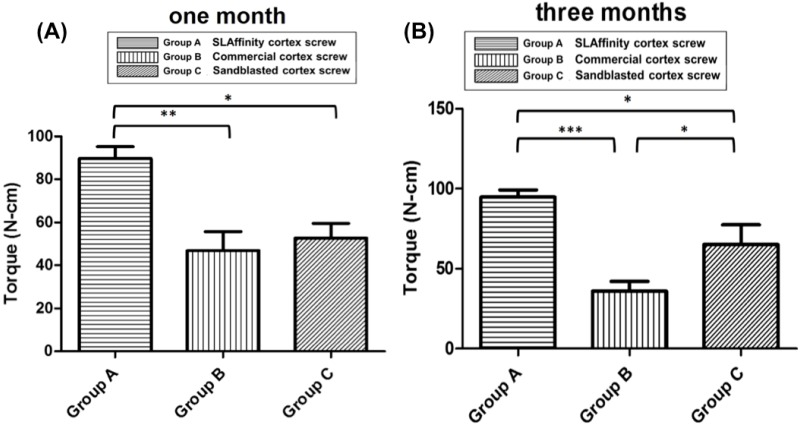
Peak insertional torques of different cortex screws in cortical bones after implantation. (A) 1 month and (B) 3 months after implantation. Group A: SLAffinity group, Group B: commercial group, Group C: sand-blasted group. *: *p* < 0.05,**: *p* < 0.01, and ***: *p* < 0.001.

**Fig 9:**
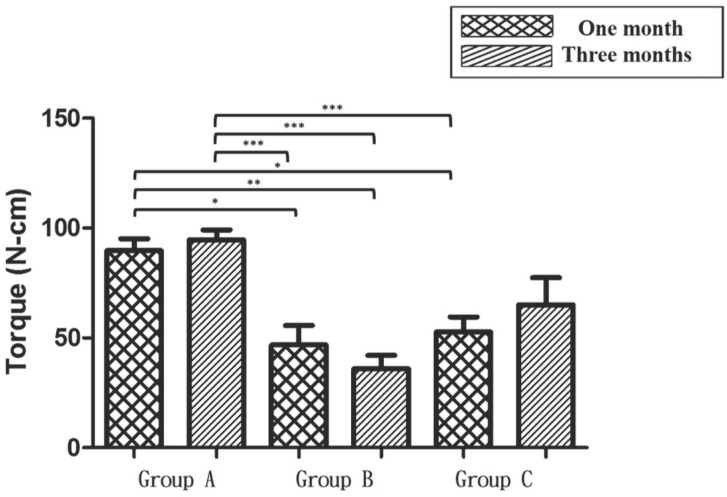
Comparison of peak insertional torques of different cortex screws in cortical bones after implantation. Group A: SLAffinity group, Group B: commercial group, Group C: sand-blasted group. *: *p* < 0.05,**: *p* < 0.01, and ***: *p* < 0.001.

The RTVs of cortical screws at 1 and 3 months are displayed in Fig 8A and 8B, respectively. At 1 month, the SLAffinity group showed a higher mean RTV (89.64 N·cm) than those of the sand-blasted (52.72 N·cm; *p* = 0.019) and commercial groups (46.84 N·cm; *p* = 0.01). The mean RTVs of the SLAffinity, sand-blasted, and commercial groups at 3 months were 94.67, 65.08, and 35.99 N·cm, respectively. The SLAffinity group had a higher mean RTV than those of the the sand-blasted (*p* = 0.02) and commercial groups (*p* < 0.001). Specially, there was a statistically significant difference between the sand-blasted and commercial groups at 3 months (*p* = 0.03). Comparing the results at 1 month to 3 months, there was no significant difference in RTV between the SLAffinity (*p* = 0.998), sand-blasted (*p* = 0.913), and commercial groups (*p* = 0.931). However, an increase in RTV at 3 months was found in the sand-blasted and SLAffinity groups; the RTV of the commercial group decreased (Fig 9).

**Fig 10:**
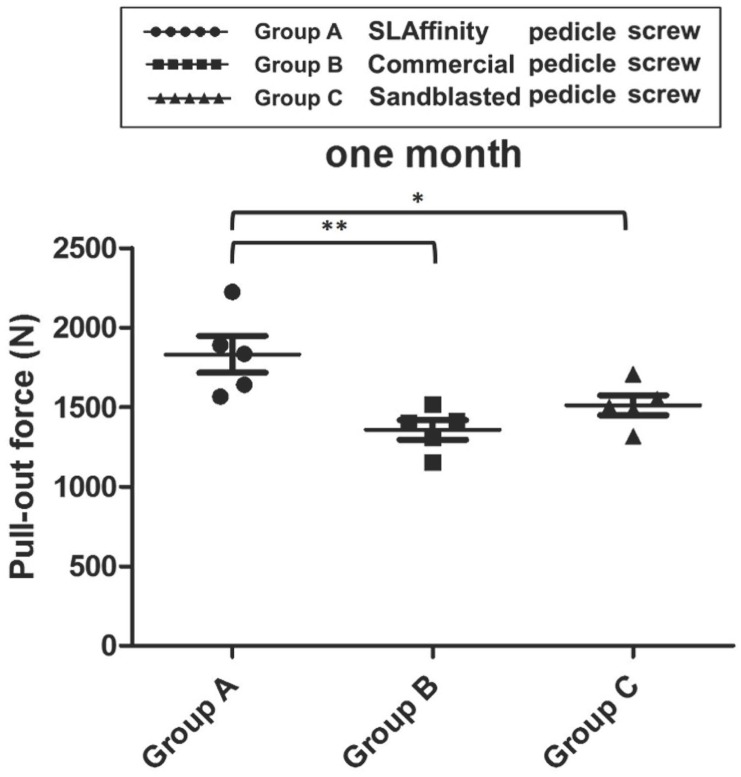
Comparison of pull-out force among different groups 1 month after implantation. *: *p* < 0.05 and **: *p* < 0.01.

**Fig 11:**
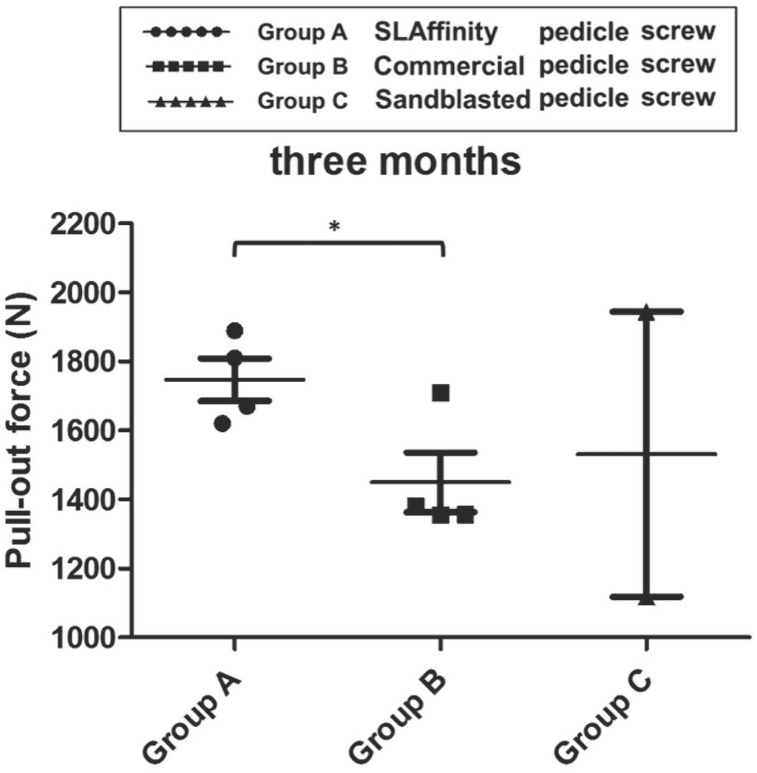
Comparison of pull-out force among different groups 3 months after implantation. *: *p* < 0.05.

In the pull-out test of pedicle screws, the pull-out strength for the screws with SLAffinity was higher than those of the sand-blasted (*p* = 0.02) and commercial screws (*p* = 0.005) 1 month after implantation. No significant difference was seen between the sand-blasted and commercial screws (*p* = 0.057) (Fig 10). In addition, the SLAffinity group showed higher pull-out strength at 3 months compared to that of the commercial group (*p* = 0.017). However, the difference between the SLAffinity and sand-blasted groups at 3 months was not statistically significant (*p* = 0.34) (Fig 11).

### Histological and histomorphometric analyses

**Fig 12:**
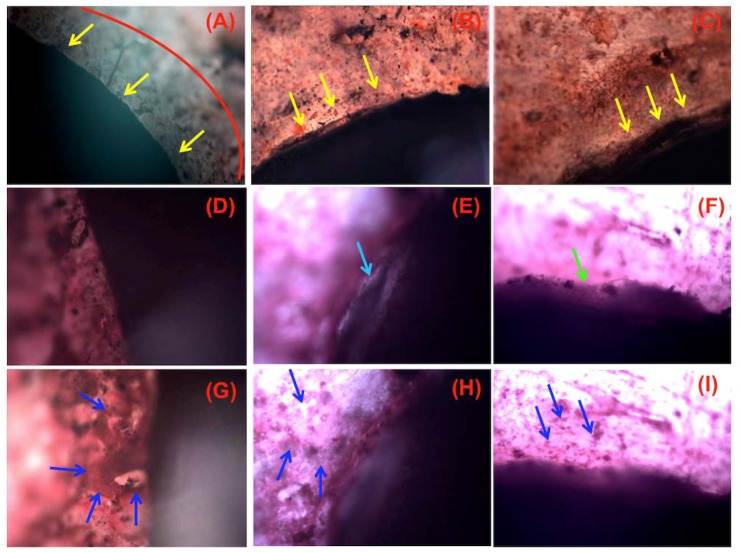
Undecalcified histological sections of peri-implant bone formation following osteointegration of different cortex screws in tibia. (A, D and G) SLAffinity, (B, E and H) commercial and (C, F and I) sand-blasted cortex screws. Red semicircle indicates new bone formation at interface between host bone tissue and screw implant. Osteocyte formation is indicated by yellow arrow. Light blue and green arrows indicate that a large gap is observed between the implant and host bone and some bony ongrowth have enlarged the contact interface with the trabecular bone, respectively. The newly bone formation in peri-implant tissues from different implants are indicated by dark blue arrows. (H&E; A, B and C, original magnification X10; D-I, original magnification X20).

**Fig 13:**
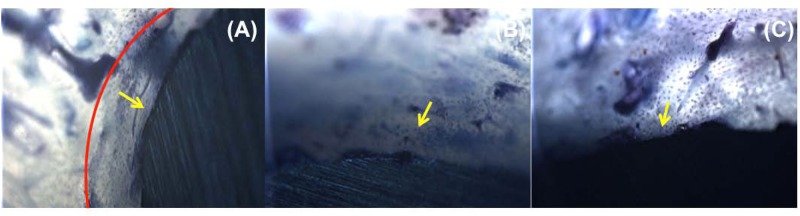
Photomicrography of slides stained by toluidine blue. Histological samples of (A) SLAffinity, (B) commercial, and (C) sand-blasted groups at 3 months post-surgery. Red semicircle indicates new bone formation at interface between host bone tissue and screw implant. Osteocyte formation is indicated by yellow arrow (original magnification X10).

The histology of the bone-implant interface of cortex screws shows that bone formation was not continuous around the screws; the degree of bone formation was surface-dependent. In other words, the screws with SLAffinity had the most bone formation at the bone-implant interface 3 months after implantation (Fig 12A). The SLAffinity group demonstrated that new calcified bone traculae are in tight contact with the implant surface and flattened cells with interposition of cuboidal cells aligned along the implant surface 3 months after implantation (Fig 12D). SLAffinity implant at 3 months also showed secondary osteons in the contact area and cement lines running perpendicular to the implant surface (Fig 12G). Moreover, the sand-blasted group presented greater osseous formation and integration than those of the commercial group (Fig 12C). Bone remodeling in the cortical area and bony ongrowth along the contact interface with the trabecular bone could be observed in sand-blasted group 3 months after implantation (Fig 12F and 12I). For commercial group, new bone trabeculae can be seen around the implant where a large gap was observed between the implant and host bone (Fig 12E). The loss of bone mass and disintegration of bone structure were also found in commercial group (Fig 12H). Although some bone contact was found in the commercial group, the areas were also covered by fibrous tissue (Fig 12B). During histological processing, the fibrous tissue contracted and detached from the surface. Fibrous tissues were also seen adjacent to SLAffinity or sand-blasted surfaces, but the fibrous layer was thinner and the gap was smaller due to processing dehydration. On most SLAffinity or sand-blasted surfaces, cells were directly attached to the surface and had produced a mineralized matrix (Fig 12(A), 12(D), 12(C) and 12(F), respectively). Furthermore, bone cells in the peri-implant bone formation of the screws were verified by toluidine blue staining. The results indicate that the screw with SLAffinity produced substantial osseous tissue and contained plenty of osteoblasts around the screw compared to those in other groups (Fig 13A–13C).

## Discussion

The processes of osseointegration involve physical mechanics and cell biology, which is quite complex. Many factors influence the formation and maintenance of bone at the implant surface. The rate and quality of osseointegration for titanium implants are related to surface properties. Surface composition, hydrophilicity, roughness, and waviness may play a role in bone-implant interaction and osseointegration [21]. In the present study, we found that screws with a SLAffinity surface had a greater RTV for cortical screws and a higher pull-out strength of pedicle screws compared to those for screws with a sand-blasted surface and commercially available screws 3 months after implantation. This study also demonstrated that the most lengths of pedicle screws with a SLAffinity surface were allocated in bone, which is greater than those for sand-blasted surface and commercial screws 3 months after implantation (S3 Fig). Moreover, the present study showed that the bone volume of implant inserted into bone and the percentage of implant inside bone tissue increased over time, which can be interpreted as signs of on-going bone remodeling at the bone–implant interface in SLAffinity group. These results plausibly explain the increased mechanical strength observed in the SLAffinity group. From histological analysis, more bone tissue and osteoblasts formed around the SLAffinity screws, which had a rough microarchitecture, leading to a higher removal torque force. Furthermore, the part of the osteoblasts location difference seen between cells grown on the commercial, sand-blasted, and SLAffinity substrates is due to the chemistry of the surface. These results indicate that sand blasting has certain effects on osteoblasts, but in combination with acid etching, there is a synergistic increase in cell differentiation and tissue formation. In other words, SLA produces a complex surface with both microroughness and waviness and thus is an efficient method for modifying the surface structure of screws, regulating osteoblast phenotype, and augmenting bone formation. Our previous study showed that SLAffinity-treated implant surfaces were superimposed on a nanoporous structure after electrochemical anodization, and that the pore size of this structure was controlled in the range of 70 to 90 nm on the specimens, as compared to SLA-treated and untreated implants, providing a three-dimensional biomimetic environment for osteocyte growth [16]. The surface hydrophilicity of a material can promote bone mineralization and enhance rapid osseointegration. For this reason, the SLAffinity-treated implant had good oxide chemistry and wettability, which can enhance cell attachment and proliferation, as well as osteoblastic differentiation. This is due to the TiO_2_ surface of this implant being more hydrophilic than SLA-treated and untreated implants, respectively [16]. In the present study, the cortex screws with a SLAffinity surface achieved the greatest amount of bone contact in cancellous bone after 3 months of healing, as compared to sand-blasted and commercial groups. Additionally, compared to the other groups, new bone trabeculae are in tight contact with the implant surface and many cuboidal osteoblasts lining on the osteoid tissue, and gap is not seen between the implant surface and host tissue in SLAffinity group. Most importantly, SLAffinity-treated screws could result in secondary osteons in the contact area and cement lines running perpendicular to the implant surface indicated the occurrence of cortical bone remodeling. On the other hand, previous study documents that titanium implants with a SLA surface have superior bone apposition in the area of bone-implant interface during healing compared with a TPS surface in mandibular bone [22]. In our present study, we also found that new bone deposition could occur on the SLAffinity and sand-blasted surfaces while in the case of commercial group for bone deposition was restricted to gaps around the bone-implant interface. Therefore, this study revealed that the SLAffinity group had enhanced osteoblast growth and osteoid tissue formation along the bone-screw interface, indicating a perfect osseointegration.

Bone healing around implants involves a cascade of cellular and extracellular biological events that take place at the bone-implant interface until the implant surface appears finally covered with a newly formed bone [23]. The essential biological factor to come into contact with an endosseous implant is blood. This cascade of biological events of bone formation is regulated by growth and differentiation factors released by the activated blood cells at the bone-implant interface [24]. The blood compatibility of implants with an oxide film obviously improves as the thickness of the titanium oxide layers increases. This means that oxide formation on implant surfaces can enhance biocompatibility and hemocompatibility. A previous study demonstrated that a SLAffinity specimen had an obviously thicker oxide layer (~500 nm) on the surface. The specific surface characteristics of a SLAffinity specimen improved wettability and promoted cell proliferation, adhesion, and spreading [14, 17]. In the present study, the SLAffinity group presented a larger area of the bone-screw interface, as well as more osteogenic cells and new bone formation at the peri-implant tissue, which may result from the superior surface properties of this implant. As noted above, surface chemistry is likely to have contributed to the outcome, as adsorption of serum components might impact the types of cells that attach to screws and their production of local regulatory factors [25]. Moreover, the SLAffinity group showed greater mechanical strength, which may reflect the importance of the area of the bone-screw interface at the peri-implant bone in osseointegration. Currently, an implant is considered as being osseointegrated when there is no progressive relative movement between the implant and the bone with which it has direct contact. This study showed that SLAffinity specimens presented a larger area of the bone-screw interface at the peri-implant bone compared with those on commercial and sand-blasted specimens, which means that the implant with a SLAffinity surface can easily attract and stimulate osteoblasts in direct contact with the implant surface; these osteoblasts eventually remodel into lamellar bone (osseointegration). Even though SLA has been widely used in dental implants in recent years and shows promising outcomes, this surface treatment has not been applied to pedicle screws, which require bone integration at the bone-implant interface for success. This study proves that SLAffinity-treated screws improve osteoconductivity compared to current commercial smooth surfaced screws.

Cortex or pedicle screw fixation is one of the most commonly used surgical methods to restore stability to fractured or unstable tibia or spine caused by traffic accidents or sports injuries. For internal fixation, cortex screws have greater resistance to screw pull-out than a screw in adjacent trabecular bone. Pedicle screw fixation better prevents motion in each translational plane of motion and the rotational axis, with minimal risk to the neural constituents when properly applied. The strength of fixation is primarily determined by the mechanical and material properties of the cortex or pedicle screw, as well as the biomechanical properties of the bone-screw implant interface. In addition, the surface modification method applied to a screw is an important element for augmenting osseous fixation and integration. Screw implants with different surface modifications that provide macrotextured surfaces, e.g., plasma spraying or hydroxyapatite coating, have better bone-to-implant contact in the late osseointegration period [26]. However, some studies have reported erosion of the hydroxyapatite layer [27] and peri-implant bone loss, resulting in a higher bone failure rate [28, 29] by implants with a hydroxyapatite surface. Moreover, a previous study found that implants with SLA surfaces had higher bone-to-implant contact percentages than those of implants with plasma-sprayed surfaces [30]. These results indicate that a roughened surface itself (titanium with a plasma-sprayed surface) does not stimulate early bone-implant integration. In this study, a titanium screw surface was sand-blasted with large particles, creating a significantly rough surface, and was then acid-etched, forming a finely rough and microtextured surface. The SLA surface texture improves the initial implant stability in bone of low density and increases the quality of the bone-to-implant interface [31]. This method of surface modification can efficaciously reduce the probability of surface contamination and of micro-particle dissemination into the surrounding tissues [32].

In this study, we evaluated screws with a SLAffinity surface. The screws combine the main properties of a roughened titanium surface: microroughness and high waviness, which favor bone cell differentiation and osseointegration. A previous study showed that SLA implants had higher mean RTVs than those of machined surface (MA) implants [10, 29, 33–35] and that RTV increased with time [11]. However, a recent study indicated that the RTVs of SLA implants were not significantly different compared to those of MA implants at 0 and 8 weeks [36]. The study ascribed this finding to the use of rabbit femoral condyle instead of the tibia as an implant model. The bone density and thickness in the two regions are different. Another study showed that the RTVs of SLA implants were not statistically different compared to those of MA implants in a beagle model after mechanical loading [37]. Here, the porcine tibia and spine model was chosen because no previous studies on bone internal fixation and osseous integration examined the effect of SLAffinity implants. Therefore, the results from the present study may be useful for pre-clinical testing.

There were some limitations in this study in the radiographic analysis and biomechanical experiment of pedicle screws. In the radiographs, the limitations of the resolution of the implant-bone interface from the metal scatter by micro-CT interfered with the quantity assessment of the tissue ingrowth at the interface. For the mechanical test, the applied loading may not exactly represent the physiological loading for pedicle screws. However, pull-out tests have been used extensively to measure biomechanical holding power and gauge the amount of stability that has been attained [38]. Although the screw direct pull-out is not the clinical failure mode, pull-out testing is still considered a good predictor of pedicle screw fixation strength [39]. The biomechanical environment in the current study cannot be completely reconstructed as the actual physiological situation, but this analytical procedure can still provide favorable information for reference. In addition, the influence of the SLAffinity implants on the tibia and spine for long-term in vivo implantation has not yet been completely verified. This experiment is important to conduct before clinical implementation.

## Conclusion

The present study showed that implants with novel SLA surface modification (SLAffinity) not only increase RTV and pull-out strength but also enhance osteoblast production, cell matrix excretion, and bone formation at the bone-implant interface compared to those of implants with a sand-blasted surface and commercially available screws. Therefore, SLAffinity implants may be ideal implants for better bone integration, especially for patients with fracture or osteoporosis.

## Supporting information

S1 Fig Appearances of three different types of pedicle screw used in study. (TIFF)

S2 Fig Three types of pedicle screw inserted into vertebral body 3 months after implantation. (TIF)

S3 Fig Length of pedicle screws that were at least allocated in bone 3 months after implantation. (TIF)
