# Supplementary figures and images for: Evaluation of osseous integration of titanium orthopedic screws with novel SLA treatment in porcine model
Source: PLoS One. 2017 Nov 17;12(11):e0188364. doi: 10.1371/journal.pone.0188364 (PMC5693293; doi:10.1371/journal.pone.0188364)

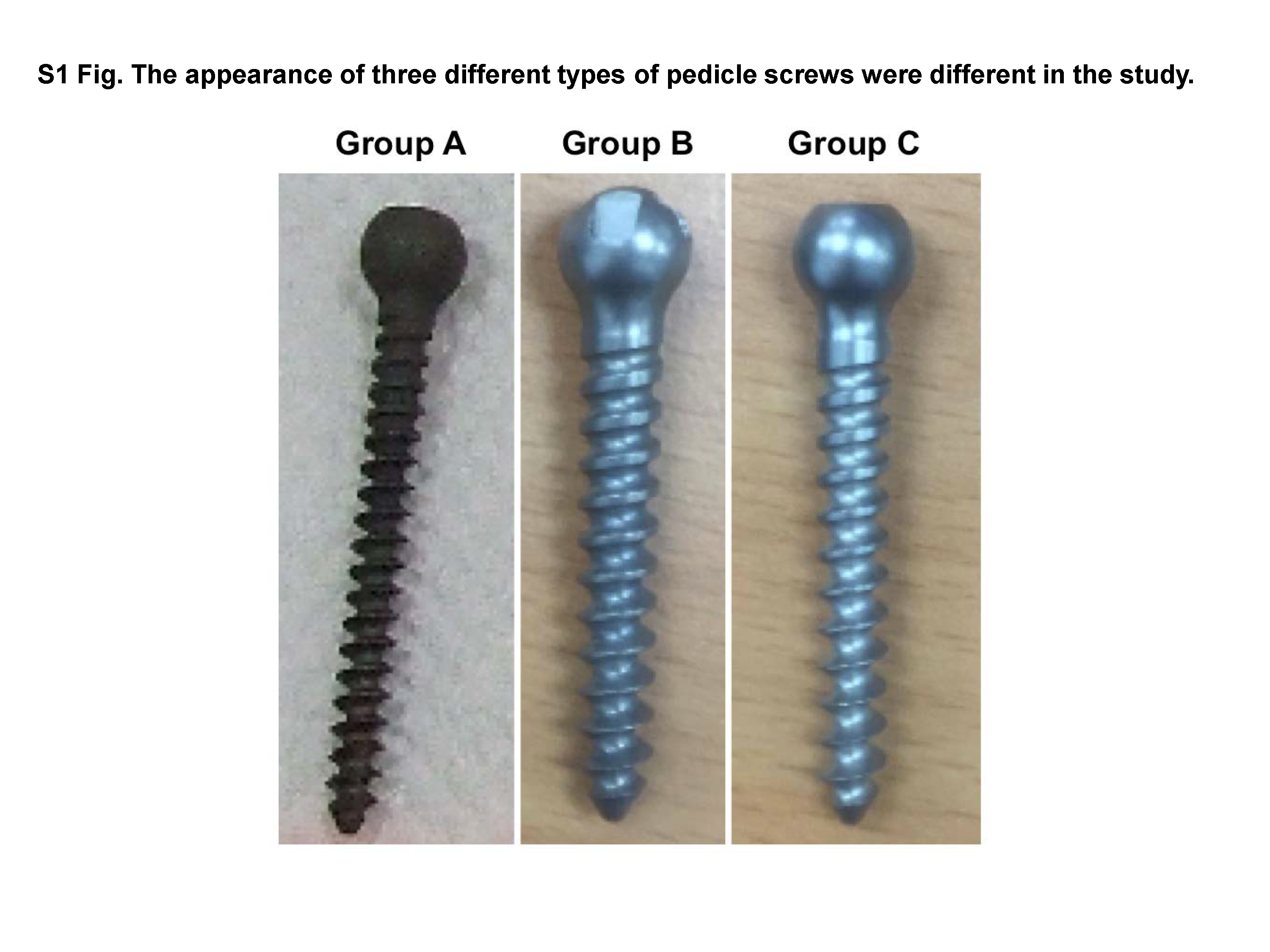

Supplement: S1 Fig — (TIFF) [file pone.0188364.s001.tiff]

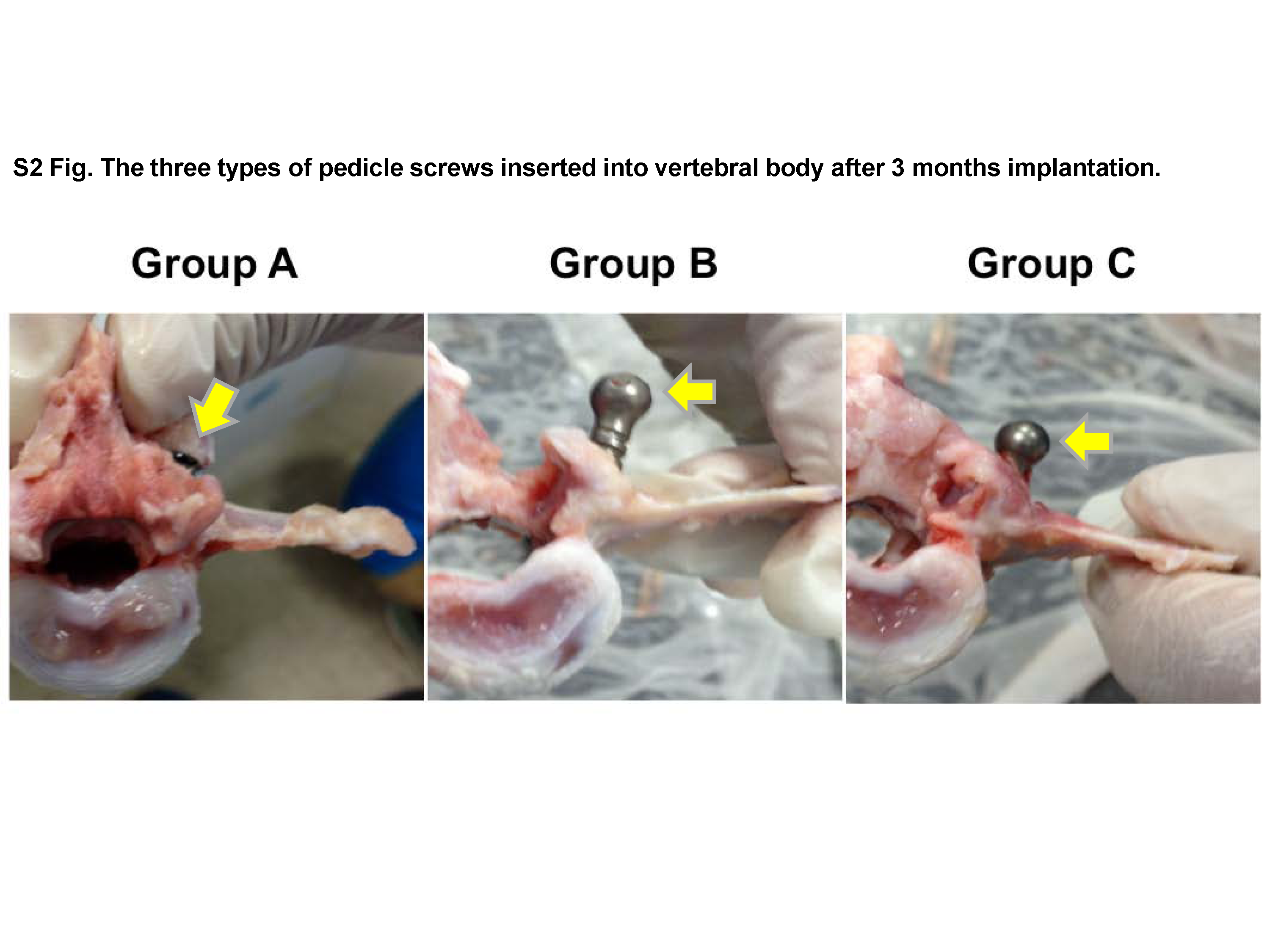

Supplement: S2 Fig — (TIF) [file pone.0188364.s002.tif]

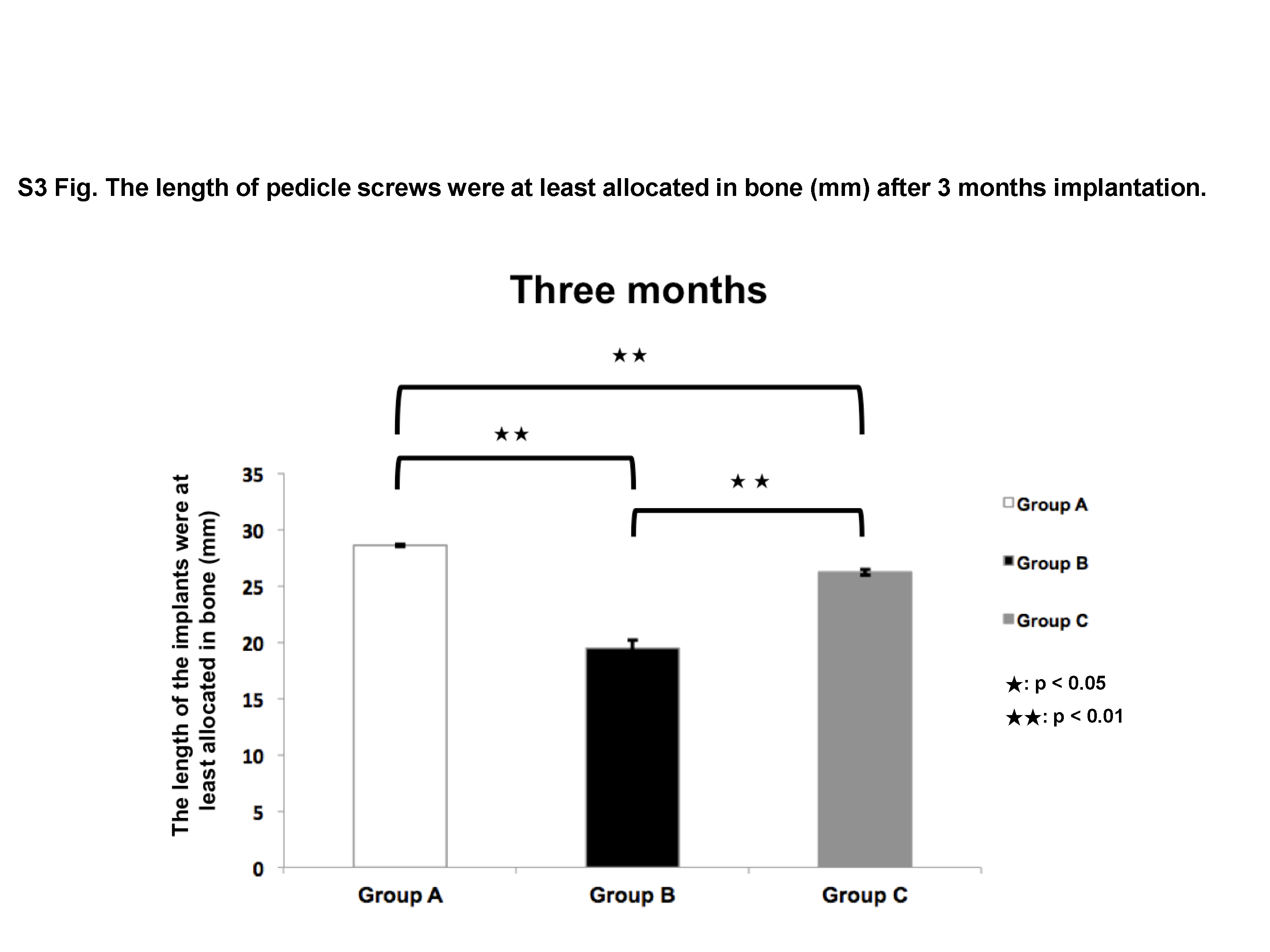

Supplement: S3 Fig — (TIF) [file pone.0188364.s003.tif]
